# Supplementary material for: Content-rich biological network constructed by mining PubMed abstracts
Source: BMC Bioinformatics. 2004 Oct 8;5:147. doi: 10.1186/1471-2105-5-147 (PMC528731; doi:10.1186/1471-2105-5-147)
Supplement: Additional File 2 — The original results of the above study (non-essential files are deleted to keep the file size under the limit set by BMC bioinformatics). [file 1471-2105-5-147-S2.bz2 › chilibotAdditionalFile2/dip05/764ID9732274E18153/html/DNAK_RNE.html]

 


 **DNAK** and **RNE** 
  
Found 1 abstracts in PubMed, retrieved 1.  
 

 What does Google say? 
 PDF only 
| .edu only 

---

**Interactive relationship** (e.g. stimulation, inhibition, etc)

**Non-interactive relationship** (e.g. studied together, co-existance, homology, etc.)

- N terminal sequencing of these proteins revealed the presence in the RNase E  [ **RNE** ]  complex not only of polynucleotide phosphorylase but also of  **DnaK** , RNA helicase, and enolase.  Ref: 8632981 Proc Natl Acad Sci U S A, 1996
